# Comorbidity profile in dementia with Lewy bodies versus Alzheimer’s disease: a linkage study between the Swedish Dementia Registry and the Swedish National Patient Registry

**DOI:** 10.1186/s13195-014-0065-2

**Published:** 2014-10-06

**Authors:** Seyed-Mohammad Fereshtehnejad, Soheil Damangir, Pavla Cermakova, Dag Aarsland, Maria Eriksdotter, Dorota Religa

**Affiliations:** 1 Division of Clinical Geriatrics, Department of Neurobiology, Care Sciences, and Society, Center for Alzheimer Research, Karolinska Institutet, Stockholm, Sweden; 2 Division of Neurogeriatrics, Department of Neurobiology, Care Sciences, and Society, Center for Alzheimer Research, Karolinska Institutet, Stockholm, Sweden; 3 International Clinical Research Center and St. Anne’s University Hospital, Brno, Czech Republic; 4 Centre for Age-Related Diseases, Stavanger University Hospital, Stavanger, Norway; 5 Department of Geriatric Medicine, Karolinska University Hospital, Stockholm, Sweden

## Abstract

**Introduction:**

Compared to Alzheimer’s disease (AD), dementia with Lewy bodies (DLB) is usually associated with a more complex clinical picture and higher burden of care. Yet, few investigations have been performed on comorbidities and risk factors of DLB. Therefore, we aimed to compare clinical risk factors and comorbidity profile in DLB and AD patients using two nationwide registries.

**Methods:**

This is a linkage study between the Swedish dementia registry (SveDem) and the Swedish National Patient Registry conducted on 634 subjects with DLB and 9161 individuals with AD registered during the years 2007–2012. Comorbidity profile has been coded according to the International Classification of Diseases version 10 (ICD 10) in addition to the date of each event. The main chapters of the ICD-10, the Charlson score of comorbidities and a selected number of neuropsychiatric diseases were compared between the DLB and AD groups. Comorbidity was registered before and after the dementia diagnosis.

**Results:**

“*Mental and behavioral disorders”, “diseases of the nervous system”, “diseases of the eye and adnexa”*, diseases of the “*circulatory*”, “*respiratory*”, and “*genitourinary*” systems, “*diseases of the skin and subcutaneous tissue*” and “*diseases of the musculoskeletal system and connective tissue*” occurred more frequently in the DLB group after multivariate adjustment. Depression [adjusted OR = 2.12 (95%CI 1.49 to 3.03)] and migraine [adjusted OR = 3.65 (95%CI 1.48 to 9.0)] were more commonly recorded before the diagnosis of dementia in the DLB group. Following dementia diagnosis, ischemic stroke [adjusted OR = 1.89 (95%CI 1.21 to 2.96)] was more likely to happen among the DLB patients compared to the AD population.

**Conclusions:**

Our study indicated a worse comorbidity profile in DLB patients with higher occurrence of depression, stroke and migraine compared with the AD group. Deeper knowledge about the underlying mechanisms of these associations is needed to explore possible reasons for the different pattern of comorbidity profile in DLB compared to AD and their prognostic significance.

## 1 Introduction

Dementia with Lewy bodies (DLB) is the second most common type of degenerative dementia after Alzheimer’s disease (AD) [1]. Compared with AD, DLB is associated with poorer prognosis, shorter time to nursing home admission, higher care burden and higher healthcare costs [2]-[4]. However, cognitive and functional decline do not seem to differ between these two groups [5]. In addition to a more complex clinical picture of DLB that is characterized by visual hallucinations, motoric symptoms, sleep disorders and autonomic dysfunction, comorbidities may be crucial predictors of worse outcomes in DLB patients. Comorbid diseases significantly increase utilization of healthcare resources [2], predict key outcomes of acute hospital care in older people [6] and are associated with a higher risk of death after dementia diagnosis [7].

Little attention has been paid to research on comorbidities and risk factors of DLB. Previous studies showed that occurrence of depression [8],[9] and anxiety [10] is higher in DLB patients when compared with AD. Depression has been suggested as a risk factor for DLB [9] and was more likely to persist over time in DLB subjects compared with AD patients [11]. History of stroke and anxiety has been reported to occur more often in DLB patients than in healthy controls, but not in comparison with AD patients [9].

Correct assessment of risk factors and comorbidities is a central part of clinical management of DLB patients. Furthermore, understanding them may provide new insights into the underlying pathophysiology of DLB. In this study, capitalizing on two nationwide registries, we compared clinical risk factors and comorbidity profiles for AD and DLB in 9,795 subjects, including 634 patients with DLB. To the authors’ best knowledge this is one of the largest published DLB cohorts.

## 2 Methods

This study was performed by linking the Swedish Dementia Registry (SveDem) and the Swedish National Patient Registry. The personal identity number was used as the unique identifier for merging the two databases. Patients were followed-up until 31 December 2012.

### 2.1 Swedish Dementia Registry

SveDem is a nationwide registry that includes newly diagnosed dementia patients from Sweden [12]. SveDem is a web-based quality registry, initiated in 2007, to improve the quality of diagnostics work-up, treatment and care of dementia across the country [13]. The majority of patients are registered through specialist care units (memory clinics) and the type of dementia is recorded as early-onset AD, late-onset AD, mixed AD, vascular dementia, DLB, frontotemporal dementia, Parkinson’s disease with dementia, unspecified dementia or others.

At the end of 2012, 58 specialist units (93% of all in Sweden) and 659 primary care centers (60% of all in Sweden) were affiliated with SveDem. Using an estimated incidence rate of 20,000 patients that develop dementia in Sweden each year [14], the approximate coverage of incident dementia cases in SveDem in 2012 was 36%. However, the study population represents a census of all newly diagnosed dementia patients because all of those who referred to the registered centers were recruited.

This study population comprised only two subgroups, 634 individuals with DLB (DLB group) and 9,161 cases with AD (AD group), recruited during the years 2007 to 2012. Dementia was diagnosed according to the International Classification of Diseases (ICD) version 10 criteria [15]. In addition, McKeith criteria were used to diagnose DLB [16].

Information about age, sex, living conditions, medication profile, diagnosis of DLB and AD (ICD version 10 codes of G31.8 for DLB and G30.0 and G30.1 for AD), baseline Mini-Mental State Examination (MMSE) score [17] and date of referral for dementia work-up were obtained from the SveDem records.

### 2.2 Swedish National Patient Registry

The Swedish National Patient Registry is administered by the Swedish National Board of Health and Welfare, which covers inpatient care in Sweden to 1987 [18]. The doctor responsible for the patient determines the diagnoses in the registry based on clinical evaluations and laboratory assessments on that particular occasion of in-hospital care. Diagnoses were coded using the latest version of the ICD according to the year of registration.

At the time of discharge, the physician registers a main diagnosis and one or more secondary diagnoses if applicable for each patient. A similar procedure is also performed through all outpatient clinics and afterwards. The diagnostic codes are sent by the hospitals and outpatient clinics to the National Board of Health and Welfare. Later, one main diagnosis and up to seven secondary diagnoses are registered together with demographics and administrative information such as hospital, clinic, dates of admission and discharge, surgical procedures and patient characteristics including age, sex and place of residence. Data obtained from 2000 to 2012 were used for this study.

### 2.3 Comorbidity profile

In addition to the main chapters of the ICD version 10, another comparison was performed based on the comorbidity scoring of the ICD version 10 codes recommended by the Royal College of Surgeons called the Charlson Score [19]. The total Charlson score of comorbidities was calculated by counting the number of comorbidity categories without any preassigned weights [20]. Data on a selected list of neuropsychiatric diseases were also extracted from the Swedish National Patient Registry using the ICD version 10 codes as follows: depression (F32, F33), anxiety (F40, F41), behavioral disorders (F07, F10, F11, F12, F13, F14, F15, F16, F17, F18, F19, F53, F54, F59, F66, F68, F69, F98, R46), bipolar affective disorder (F31), sleep disorder (F51, G47), syncope (R55), ischemic stroke (I63, I64, I67, I69), cerebral hemorrhage (I60, I61, I62), epilepsy (G40), migraine (G43) and other types of headache (G44, R51). The time of registration was used to check whether the disease occurred before or after dementia diagnosis as well as to calculate the time intervals between the dates.

Since no information is available on the exact time when the symptoms started, we used the date of registration as the initial time point for dementia and the comorbidities. Therefore, if the date of registration for any of the comorbidities has been recorded prior to the date of registration in SveDem, we considered that comorbidity has occurred before dementia, and *vice versa*.

### 2.4 Statistical analysis

The mean (standard deviation (SD)) and frequency percentage (%) were reported to describe quantitative and categorical variables. For univariate comparisons, the chi-square statistic and Fisher’s exact test were used to compare the relative frequency of nominal variables (that is, sex, comorbidities) between the two study groups where appropriate. To compare the mean value of quantitative variables between the DLB and AD groups, either an independent-sample *t* test or a Mann–Whitney *U* test were used for normally or skewed distributions, respectively.

Further multivariate analysis was performed to adjust for the confounding effect of the baseline differences in age and sex between the DLB and AD groups. For this purpose, a binary logistic regression model was applied to evaluate the differences observed in the prevalence of different comorbidities between the DLB and AD groups adjusted for the baseline confounders. Afterwards, the adjusted odds ratio (OR) and its corresponding 95% confidence interval (CI) for each comorbidity were calculated. For the comorbidities that occurred before the date of referral for dementia, type of dementia (DLB vs. AD) was considered the dependent variable. If the comorbid event happened after dementia diagnosis, the comorbidity was defined as the dependent variable and the type of dementia as a predictor in the multivariate model. In all analytical procedures, two-tailed *P* < 0.05 was considered to show statistically significant difference. Data were analyzed using SPSS software version 22 (IBM Co., Chicago, IL USA).

### 2.5 Ethical issues

The regional Ethical Committee of Stockholm approved data collection (Drn. 2013/147-31/2), as well as the merging and the analytical procedures performed in this study. The patients were informed orally and in writing about SveDem and could decline participation. Data were coded and anonymized before statistical analysis.

## 3 Results

### 3.1 Baseline characteristics

A total number of 634 individuals with DLB and 9,161 AD patients were recruited. The DLB group consisted of 382 (60.3%) males and 252 (39.7%) females with a mean age of 76.5 (SD = 7.1) years*.* The AD group comprised 3,188 (34.8%) males and 5,973 (65.2%) females with a mean age of 77.6 (SD = 8.3) years at the time of dementia diagnosis. Results of the Pearson chi-square test and the independent-samples *t* test revealed that there were significantly more males (*P* < 0.001) and fewer old patients (*P* < 0.001) in the DLB group. Other baseline, demographic and medication characteristics of the two study groups are presented and compared in Table 1. The mean of the baseline MMSE score was quite similar in the DLB (21.4 (SD = 5.0)) and AD (21.5 (SD = 5.0)) groups (*P* = 0.593). However, in the DLB group the proportion of patients in nursing homes was larger than that in the AD group (11.8% vs. 5.6%, *P* < 0.001).

**Table 1:** Baseline, diagnostic and medication characteristics of the two study groups: cases suffering from dementia with Lewy bodies versus Alzheimer’s disease patients

**Characteristic**	**Dementia with Lewy bodies**	**Alzheimer’s disease**	***P*** **value**
	**(*n*** **= 634)**	**(*n*** **= 9,161)**	
Gender			**<0.001**^a^
Female	252 (39.7%)	5973 (65.2%)	
Male	382 (60.3%)	3188 (34.8%)	
Age (years)	76.5 (7.1)	77.6 (8.3)	**<0.001**^b^
MMSE score	21.4 (5.0)	21.5 (5.0)	0.593^b^
Body mass index (kg/m^2^)	24.4 (4.1)	24.3 (4.1)	0.565^b^
Living place			**<0.001**^a^
Own house	559 (88.2%)	8627 (94.4%)	
Nursing home	75 (11.8%)	512 (5.6%)	
Co-resident			**<0.001**^a^
Yes	218 (36.0%)	3879 (44.0%)	
No	387 (64.0%)	4938 (56.0%)	
Medication (at the time of diagnosis)			
Cholinesterase inhibitors	465 (73.6%)	6598 (72.7%)	0.272^a^
NMDA antagonist	95 (15.0%)	895 (9.9%)	**<0.001**^a^
Antidepressants	220 (34.8%)	2382 (26.3%)	**<0.001**^a^
Antipsychotics	103 (16.3%)	447 (4.9%)	**<0.001**^a^
Anxiolytics	84 (13.3%)	747 (8.2%)	**<0.001**^a^
Hypnotics	106 (16.8%)	1292 (14.2%)	0.065^a^
Cardiovascular drugs	365 (57.8%)	4825 (53.2%)	0.054^a^
Total number of drugs	4.7 (3.0)	3.7 (2.9)	**<0.001**^b^

Data presented as number (%) or mean (standard deviation). Statistically significant differences (*P* < 0.05) in bold. MMSE, Mini-Mental State Examination; NMDA, *N*-methyl d-aspartate. ^a^Pearson chi-square statistic. ^b^Independent-sample *t* test.

DLB patients were under treatment with a significantly higher number of medications (4.7 (SD = 3.0)) compared with the AD group (3.7 (SD = 2.9)). A multivariate Poisson regression model showed that DLB patients received a higher number of drugs (*B* = 0.268 (95% CI = 0.230 to 0.307), *P* < 0.001) after adjustment for sex and age. While a similar proportion of patients were treated with cholinesterase inhibitors in both the DLB (73.6%) and AD (72.7%) groups (*P* = 0.272), *N*-methyl d-aspartate antagonists (15.0% vs. 9.9%), antidepressants (34.8% vs. 26.3%), antipsychotics (16.3% vs. 4.9%) and anxiolytics (13.3% vs. 8.2%) were significantly more often prescribed among the DLB group.

### 3.2 International Classification of Diseases version 10 chapters

Table 2 summarizes the comorbidity profile of the DLB and AD patients based on the chapters of the ICD version 10 coding system. ‘Mental and behavioral disorders’ (66.1%) and ‘diseases of the eye and adnexa’ (57.9%) were the most common categories recorded for the DLB group apart from the ‘diseases of the nervous system’; while among the AD patients ‘diseases of the eye and adnexa’ (47.9%), ‘diseases of the musculoskeletal system and connective tissue’ (40.7%) and ‘diseases of the circulatory system’ (40.3%) were most commonly recorded as comorbid conditions. ‘Mental and behavioral disorders’, ‘diseases of the nervous system’ , ‘diseases of the eye and adnexa’ , diseases of the ‘circulatory’ , ‘respiratory’ , ‘digestive’ and ‘genitourinary’ systems and the ‘diseases of the skin and subcutaneous tissue’ occurred more commonly in the DLB group based on the univariate comparisons.

**Table 2:** Comorbidity profile of the patients with dementia with Lewy bodies versus Alzheimer’s disease patients based on the chapters of the International Statistical Classification of Diseases and Related Health Problems, 10th Revision

**Chapter**	**Blocks**	**Title**	**DLB**	**AD**	**Unadjusted**	***P*** **value**^**a**^	**Adjusted**	***P*** **value**^**b**^
			**(*n*** **= 634)**	**(*n*** **= 9,161)**	**OR (95% CI)**		**OR (95% CI)**	
I	A00 to B99	Certain infectious and parasitic diseases	87 (13.7)	1,064 (11.6)	1.21 (0.96 to 1.53)	0.111	1.25 (0.98 to 1.59)	0.069
II	C00 to D48	Neoplasms	203 (32.0)	2,733 (29.8)	1.11 (0.93 to 1.32)	0.245	1.06 (0.88 to 1.27)	0.541
III	D50 to D89	Diseases of the blood and blood-forming organs and certain disorders involving the immune mechanism	29 (4.6)	433 (4.7)	0.97 (0.66 to 1.42)	0.861	1.01 (0.68 to 1.49)	0.976
IV	E00 to E90	Endocrine, nutritional and metabolic diseases	79 (12.5)	1,088 (11.9)	1.06 (0.83 to 1.35)	0.661	1.16 (0.90 to 1.49)	0.239
V	F00 to F99	Mental and behavioral disorders	419 (66.1)	3,543 (38.7)	**3.09 (2.61 to 3.66)**	**<0.001**	**3.14 (2.63 to 3.75)**	**<0.001**
VI	G00 to G99	Diseases of the nervous system	558 (88.0)	6,508 (71.0)	**2.99 (2.34 to 3.82)**	**<0.001**	**2.78 (2.15 to 3.58)**	**<0.001**
VII	H00 to H59	Diseases of the eye and adnexa	367 (57.9)	4,390 (47.9)	**1.49 (1.27 to 1.76)**	**<0.001**	**1.69 (1.42 to 2.01)**	**<0.001**
VIII	H60 to H95	Diseases of the ear and mastoid process	96 (15.1)	1,462 (16.0)	0.94 (0.75 to 1.18)	0.586	0.97 (0.77 to 1.22)	0.810
IX	I00 to I99	Diseases of the circulatory system	293 (46.2)	3,693 (40.3)	**1.27 (1.08 to 1.49)**	**0.003**	**1.29 (1.08 to 1.52)**	**0.004**
X	J00 to J99	Diseases of the respiratory system	146 (23.0)	1,663 (18.2)	**1.35 (1.11 to 1.63)**	**0.002**	**1.30 (1.06 to 1.59)**	**0.010**
XI	K00 to K93	Diseases of the digestive system	237 (37.4)	2,966 (32.4)	**1.25 (1.05 to 1.47)**	**0.009**	1.17 (0.98 to 1.39)	0.082
XII	L00 to L99	Diseases of the skin and subcutaneous tissue	155 (24.4)	1,918 (20.9)	**1.22 (1.01 to 1.47)**	**0.036**	**1.27 (1.05 to 1.54)**	**0.016**
XIII	M00 to M99	Diseases of the musculoskeletal system and connective tissue	273 (43.1)	3,732 (40.7)	1.10 (0.93 to 1.29)	0.250	**1.19 (1.01 to 1.41)**	**0.040**
XIV	N00 to N99	Diseases of the genitourinary system	233 (36.8)	2,984 (32.6)	**1.20 (1.02 to 1.42)**	**0.030**	**1.24 (1.04 to 1.47)**	**0.015**
XV	O00 to O99	Pregnancy, childbirth and the puerperium	1 (0.2)	2 (0.0)	–	–	–	–
XVI	P00 to P96	Certain conditions originating in the perinatal period	0	0	–	–	–	–
XVII	Q00 to Q99	Congenital malformations, deformations and chromosomal abnormalities	5 (0.8)	57 (0.6)	1.27 (0.51 to 3.18)	0.609	1.55 (0.61 to 3.98)	0.358
XVIII	R00 to R99	Symptoms, signs and abnormal clinical and laboratory findings, not elsewhere classified	460 (72.6)	5,896 (64.4)	**1.46 (1.22 to 1.75)**	**<0.001**	**1.44 (1.19 to 1.73)**	**<0.001**
XIX	S00 to T98	Injury, poisoning and certain other consequences of external causes	369 (58.2)	4,679 (51.1)	**1.33 (1.13 to 1.57)**	**0.001**	**1.54 (1.30 to 1.83)**	**<0.001**
XX	V01 to Y98	External causes of morbidity and mortality	0	1 (0.0)	–	–	–	–
XXI	Z00 to Z99	Factors influencing health status and contact with health services	523 (82.5)	6,718 (73.3)	**1.71 (1.39 to 2.11)**	**<0.001**	**1.73 (1.39 to 2.16)**	**<0.001**

Adjustment is made for age, sex, and Mini-Mental State Examination score. The AD group is considered the reference group. Statistically significant ORs (*P <* 0.05) in bold. AD, Alzheimer’s disease; CI, confidence interval; DLB, dementia with Lewy bodies; OR, odds ratio. ^a^Chi-square statistics. ^b^Binary logistic regression model.

As shown in Table 2, all univariate significant differences remained statistically significant after multivariate adjustment except for the ‘diseases of the digestive system’. Moreover, DLB patients suffered more from the ‘diseases of the musculoskeletal system and connective tissue’ after adjustment for age and sex (adjusted OR = 1.19 (95% CI = 1.01 to 1.41)). The greatest between-group differences in the ICD version 10 disease categories were observed in ‘mental and behavioral disorders’ (66.1% vs. 38.7%, adjusted OR = 3.14 (95% CI = 2.63 to 3.75)), ‘diseases of the nervous system’ (88.0% vs. 71.0%, adjusted OR = 2.78 (95% CI = 2.15 to 3.58)) and ‘diseases of the eye and adnexa’ (57.9% vs. 47.9%, adjusted OR = 1.69 (95% CI = 1.42 to 2.01)), all of which were more common in the DLB group.

Further subgroup analysis was performed to assess how patients’ gender, level of cognition and living place might affect the differences in the comorbidity profile between the DLB and AD groups. As summarized in Table 3, some comorbidity categories such as ‘mental and behavioral disorders’ and ‘diseases of the nervous system’ were significantly more common in the DLB group among all of the subgroups regarding gender, cognition and living place (all *P* < 0.05). On the other hand, ‘diseases of the circulatory system’ was significantly more prevalent in the DLB patients who were female (46.8% vs. 38.6%, *P* = 0.009), had MMSE < 22 (49.6% vs. 39.1%, *P* = 0.001) and live in their own house (46.3% vs. 40.2%, *P* = 0.005).

**Table 3:** **Comorbidity profile of patients with dementia with Lewy bodies (*n*** **= 634) versus Alzheimer’s disease patients (*n*** **= 9,161) within different subgroups regarding gender, cognitive level at the time of diagnosis and living place using the chapters of the International Statistical Classification of Diseases and Related Health Problems, 10th Revision**

**Title**	**Gender**	**DLB (%)**	**AD (%)**	***P*** **value**	**Cognition**	**DLB (%)**	**AD (%)**	***P*** **value**	**Living place**	**DLB (%)**	**AD (%)**	***P*** **value**
Certain infectious and parasitic diseases	Male	14.9	12.5	0.183	MMSE ≥ 22	16.3	11.2	**0.005**	Own house	14.3	11.4	**0.038**
	Female	11.9	11.1	0.703	MMSE < 22	11.9	12.2	0.855	Nursing home	9.3	15.0	0.188
Neoplasms	Male	35.6	33.8	0.494	MMSE ≥ 22	32.8	31.6	0.652	Own house	32.4	30.1	0.259
	Female	26.6	27.7	0.701	MMSE < 22	31.9	27.9	0.169	Nursing home	29.3	25.4	0.467
Diseases of the blood and blood-forming organs and certain disorders involving the immune mechanism	Male	5.2	4.8	0.687	MMSE ≥ 22	3.6	4.5	0.447	Own house	4.7	4.6	0.967
	Female	3.6	4.7	0.403	MMSE < 22	5.9	4.8	0.414	Nursing home	4.0	6.8	0.457
Endocrine, nutritional and metabolic diseases	Male	12.8	10.9	0.262	MMSE ≥ 22	10.5	11.7	0.526	Own house	12.3	11.8	0.700
	Female	11.9	12.4	0.819	MMSE < 22	15.9	11.8	**0.046**	Nursing home	13.3	13.5	0.973
Mental and behavioral disorders	Male	67.3	37.4	**<0.001**	MMSE ≥ 22	65.7	40.8	**<0.001**	Own house	65.5	38.8	**<0.001**
	Female	64.3	39.3	**<0.001**	MMSE < 22	66.7	36.0	**<0.001**	Nursing home	70.7	36.5	**<0.001**
Diseases of the nervous system	Male	91.4	72.8	**<0.001**	MMSE ≥ 22	89.5	75.5	**<0.001**	Own house	88.7	72.2	**<0.001**
	Female	82.9	70.1	**<0.001**	MMSE < 22	86.3	66.8	**<0.001**	Nursing home	82.7	52.3	**<0.001**
Diseases of the eye and adnexa	Male	56.5	44.2	**<0.001**	MMSE ≥ 22	60.2	49.0	**<0.001**	Own house	58.3	47.7	**<0.001**
	Female	59.9	49.9	**0.002**	MMSE < 22	55.6	47.0	**0.007**	Nursing home	54.7	52.0	0.660
Diseases of the ear and mastoid process	Male	15.4	17.2	0.383	MMSE ≥ 22	17.5	17.1	0.857	Own house	15.7	16.2	0.790
	Female	14.7	15.3	0.794	MMSE < 22	14.1	14.6	0.819	Nursing home	10.7	12.9	0.588
Diseases of the circulatory system	Male	45.8	43.5	0.391	MMSE ≥ 22	43.7	40.8	0.296	Own house	46.3	40.2	**0.005**
	Female	46.8	38.6	**0.009**	MMSE < 22	49.6	39.1	**0.001**	Nursing home	45.3	41.2	0.499
Diseases of the respiratory system	Male	27.7	20.5	**0.001**	MMSE ≥ 22	22.0	17.6	**0.043**	Own house	22.9	18.0	**0.004**
	Female	15.9	16.9	0.677	MMSE < 22	24.4	18.6	**0.018**	Nursing home	24.0	20.1	0.438
Diseases of the digestive system	Male	41.6	37.0	0.079	MMSE ≥ 22	36.7	34.3	0.357	Own house	37.7	32.7	**0.013**
	Female	31.0	29.9	0.721	MMSE < 22	38.9	30.3	**0.003**	Nursing home	34.7	27.9	0.229
Diseases of the skin and subcutaneous tissue	Male	24.3	20.2	0.057	MMSE ≥ 22	25.3	22.5	0.230	Own house	24.7	21.1	**0.043**
	Female	24.6	21.3	0.217	MMSE < 22	24.1	19.3	0.056	Nursing home	22.7	18.6	0.397
Diseases of the musculoskeletal system and connective tissue	Male	39.8	36.1	0.157	MMSE ≥ 22	41.3	42.9	0.555	Own house	42.9	41.0	0.371
	Female	48.0	43.2	0.132	MMSE < 22	45.6	38.5	**0.022**	Nursing home	44.0	37.5	0.280
Diseases of the genitourinary system	Male	36.4	32.1	0.093	MMSE ≥ 22	38.0	33.6	0.108	Own house	38.3	32.6	**0.005**
	Female	37.3	32.8	0.138	MMSE < 22	35.6	31.1	0.132	Nursing home	25.3	32.6	0.205
Pregnancy, childbirth and the puerperium	Male	–	–	–	MMSE ≥ 22	0.3	0	0.062	Own house	0.2	0	0.061
	Female	0.4	0	0.117	MMSE < 22	0	0.1	1	Nursing home	0	0.4	1
Certain conditions originating in the perinatal period	Male	0	0	–	MMSE ≥ 22	0	0	–	Own house	0	0	–
	Female	0	0	–	MMSE < 22	0	0	–	Nursing home	0	0	–
Congenital malformations, deformations and chromosomal abnormalities	Male	1.0	0.6	0.300	MMSE ≥ 22	0.9	0.6	0.464	Own house	0.7	0.5	0.545
	Female	0.4	0.6	1	MMSE < 22	0.7	0.4	0.349	Nursing home	1.3	2.1	1
Symptoms, signs and abnormal clinical and laboratory findings, not elsewhere classified	Male	72.8	66.9	**0.020**	MMSE ≥ 22	74.1	67.2	**0.010**	Own house	72.3	64.5	**<0.001**
	Female	72.2	63.0	**0.003**	MMSE < 22	71.5	61.3	**0.001**	Nursing home	74.7	62.7	**0.043**
Injury, poisoning and certain other consequences of external causes	Male	50.8	45.1	**0.034**	MMSE ≥ 22	59.6	49.2	**<0.001**	Own house	58.3	50.2	**<0.001**
	Female	69.4	54.3	**<0.001**	MMSE < 22	56.7	53.5	0.312	Nursing home	57.3	64.8	0.206
External causes of morbidity and mortality	Male	0	0	–	MMSE ≥ 22	0	0	–	Own house	0	0	–
	Female	0	0	–	MMSE < 22	0	0	–	Nursing home	0	0	–
Factors influencing health status and contact with health services	Male	81.4	73.0	**<0.001**	MMSE ≥ 22	84.3	76.8	**0.001**	Own house	83.2	73.8	**<0.001**
	Female	84.1	73.5	**<0.001**	MMSE < 22	81.1	69.8	**<0.001**	Nursing home	77.3	65.8	**0.047**

Statistically significant differences (*P* < 0.05) in bold. Univariate comparisons were performed using either the chi-square or Fisher’s exact test wherever appropriate. AD, Alzheimer’s disease; DLB, dementia with Lewy bodies; MMSE, Mini-Mental State Examination.

Although ‘diseases of the respiratory system’ was more commonly recorded in the DLB patients with both MMSE ≥ 22 (*P* = 0.043) and MMSE < 22 (*P* = 0.018), with regard to gender and living place the difference was significant only among the males (27.7% vs. 20.5%, *P* = 0.001) and those who live in their own houses (22.9% vs. 18.0%, *P* = 0.004). Furthermore, ‘diseases of the digestive system’ (38.9% vs. 30.3%, *P* = 0.003) and ‘diseases of the musculoskeletal system and connective tissue’ (45.6% vs. 38.5%, *P* = 0.022) were significantly higher in the DLB group compared with the AD group only among the subgroup with MMSE < 22 at the time of diagnosis.

### 3.3 Charlson comorbidity scoring

**Figure 1:**
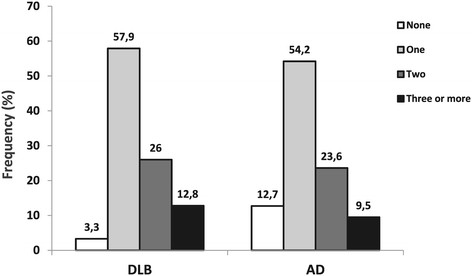
**Frequency of dementia with Lewy bodies and Alzheimer’s disease in patients with different number of comorbidities.** Frequency of dementia with Lewy bodies (DLB, *n* = 634) and Alzheimer’s disease (AD, *n* = 9,161) in patients with different number of comorbidities based on the categories of the Royal College of Surgeons Charlson Score identified on the International Classification of Disease, 10th revision codes.

Comorbidity profiles of the DLB and AD patients using the Royal College of Surgeons Charlson categorization of the ICD version 10 codes are presented in Table 4. Cerebrovascular diseases were more common in the DLB group after adjustment for age and sex (16.2% vs. 10.0%, adjusted OR = 1.74 (95% CI = 1.38 to 2.19)). As illustrated in Figure 1, 12.7% of the AD patients had a zero score according to the Charlson scoring of the comorbidities, while only 3.3% of the DLB patients showed this condition. The proportion of individuals with one, two and three or more comorbidity categories of Charlson scoring was higher in the DLB group (*P* < 0.001). Moreover, DLB patients had a significantly higher mean for the total Charlson score (1.52 (SD = 0.85) vs. 1.33 (SD = 0.89)). This difference remained significant even after multivariate adjustment (OR = 1.22 (95% CI = 1.12 to 1.33)).

**Table 4:** Comorbidity profile of the patients with dementia with Lewy bodies versus Alzheimer’s disease patients based on the Royal College of Surgeons Charlson Score indicating International Classification of Disease, 10th revision codes for 14 disease categories

**Disease category**	**DLB**	**AD**	**Unadjusted**	***P*** **value**^**a**^	**Adjusted**	***P*** **value**^**b**^
	**(*n*** **= 634)**	**(*n*** **= 9,161)**	**OR (95% CI)**		**OR (95% CI)**	
Myocardial infarction	34 (5.4)	532 (5.8)	0.92 (0.64 to 1.31)	0.643	0.85 (0.59 to 1.22)	0.372
Congestive cardiac failure	38 (6.0)	458 (5.0)	1.21 (0.86 to 1.70)	0.270	1.21 (0.85 to 1.73)	0.282
Peripheral vascular disease	15 (2.4)	271 (3.0)	0.79 (0.47 to 1.34)	0.392	0.77 (0.45 to 1.31)	0.335
Cerebrovascular disease	103 (16.2)	912 (10.0)	**1.75 (1.40 to 2.19)**	**<0.001**	**1.74 (1.38 to 2.19)**	**<0.001**
Chronic pulmonary disease	27 (4.3)	480 (5.2)	0.80 (0.54 to 1.20)	0.281	0.88 (0.59 to 1.31)	0.525
Rheumatological disease	12 (1.9)	286 (3.1)	0.60 (0.33 to 1.07)	0.081	0.64 (0.35 to 1.19)	0.160
Liver disease	4 (0.6)	48 (0.5)	1.20 (0.43 to 3.35)	0.578	1.13 (0.40 to 3.19)	0.818
Diabetes mellitus	26 (4.1)	481 (5.3)	0.77 (0.52 to 1.15)	0.206	0.73 (0.48 to 1.11)	0.145
Hemiplegia or paraplegia	0	19 (0.2)	–	0.631	–	0.998
Renal disease	11 (1.7)	98 (1.1)	1.63 (0.87 to 3.06)	0.123	1.57 (0.83 to 2.97)	0.167
Any malignancy	94 (14.8)	1283 (14.0)	1.07 (0.85 to 1.34)	0.565	0.96 (0.75 to 1.21)	0.707
Metastatic solid tumor	2 (0.3)	74 (0.8)	0.39 (0.09 to 1.59)	0.239	0.42 (0.10 to 1.74)	0.235
AIDS/HIV infection	0	1 (0.0)	–	–	–	–
Total Charlson score	1.52 (0.85)	1.33 (0.89)	**1.25 (1.15 to 1.36)**	**<0.001**	**1.22 (1.12 to 1.33)**	**<0.001**

Data presented as mean (standard deviation). Adjustment is made for age, sex, and Mini-Mental State Examination score. The AD group is considered the reference group. The dementia category of the original Charlson scoring is not reported here because the whole study population is dementia patients in our study. Statistically significant ORs (*P* < 0.05) in bold. AD, Alzheimer’s disease; CI, confidence interval; DLB, dementia with Lewy bodies; OR, odds ratio. ^a^Chi-square statistics. ^b^Binary logistic regression model.

### 3.4 Neuropsychiatric comorbidities

Considering the date of registration in SveDem and the Swedish National Patient Registry, occurrence of the selected neuropsychiatric comorbidities was determined as either before or after dementia diagnosis. As presented in Table 5, depression was the most common neuropsychiatric comorbidity before the diagnosis of dementia in both groups (6.0% in DLB and 3.0% in AD). Among strokes, cerebral infarction was more common in the DLB group than in the AD group, while the frequency of cerebral hemorrhages did not differ between the AD and DLB groups. According to univariate comparisons (Table 5), depression (*P* < 0.001), behavioral disorders (*P* = 0.012), stroke (*P* = 0.002) and migraine (*P* = 0.028) were all more frequent in individuals with DLB compared with the AD group. However, the other types of headache were not significantly different between the two groups (3.2% in DLB and 3.4% in AD, OR = 0.92 (95% CI = 0.58 to 1.45), *P* = 0.713).

**Table 5:** Occurrence and timing for different neuropsychiatric comorbidities in patients with dementia with Lewy bodies versus Alzheimer’s disease patients

**Comorbidity**	**Dementia with Lewy bodies**	**Alzheimer’s disease**	***P*** **value**^**a**^
	**(*n*** **= 634)**	**(*n*** **= 9,161)**	
Depression			**<0.001**
None	587 (92.7)	8,736 (95.5)	
Before dementia diagnosis	38 (6.0)	277 (3.0)	
After dementia diagnosis	8 (1.3)	130 (1.4)	
Anxiety			0.508
None	612 (96.7)	8,905 (97.4)	
Before dementia diagnosis	16 (2.5)	172 (1.9)	
After dementia diagnosis	5 (0.8)	66 (0.7)	
Behavioral disorders			**0.012**
None	612 (96.7)	8,964 (98.0)	
Before dementia diagnosis	9 (1.4)	107 (1.2)	
After dementia diagnosis	12 (1.9)	72 (0.8)	
Bipolar affective disorder			0.258
None	628 (99.2)	9,100 (99.5)	
Before dementia diagnosis	5 (0.8)	36 (0.4)	
After dementia diagnosis	0	7 (0.1)	
Sleep disorders			0.093
None	616 (97.3)	9,001 (98.4)	
Before dementia diagnosis	14 (2.2)	116 (1.3)	
After dementia diagnosis	3 (0.5)	26 (0.3)	
Syncope			0.472
None	587 (92.7)	8,486 (92.8)	
Before dementia diagnosis	35 (5.5)	443 (4.8)	
After dementia diagnosis	11 (1.7)	214 (2.3)	
Stroke			**0.002**
None	580 (91.6)	8,665 (94.8)	
Before dementia diagnosis	28 (4.4)	277 (3.0)	
After dementia diagnosis	25 (3.9)	201 (2.2)	
Cerebral hemorrhage			0.709
None	623 (98.4)	9,015 (98.6)	
Before dementia diagnosis	6 (0.9)	62 (0.7)	
After dementia diagnosis	4 (0.6)	66 (0.7)	
Epilepsy			0.291
None	622 (98.3)	9,022 (98.7)	
Before dementia diagnosis	8 (1.3)	66 (0.7)	
After dementia diagnosis	3 (0.4)	55 (0.6)	
Migraine			**0.028**
None	627 (99.1)	9,113 (99.7)	
Before dementia diagnosis	6 (0.9)	28 (0.3)	
After dementia diagnosis	0	2 (0.0)	

Data presented as number (%). Statistically significant differences (*P* < 0.05) in bold. ^a^Pearson chi-square statistics.

**Figure 2:**
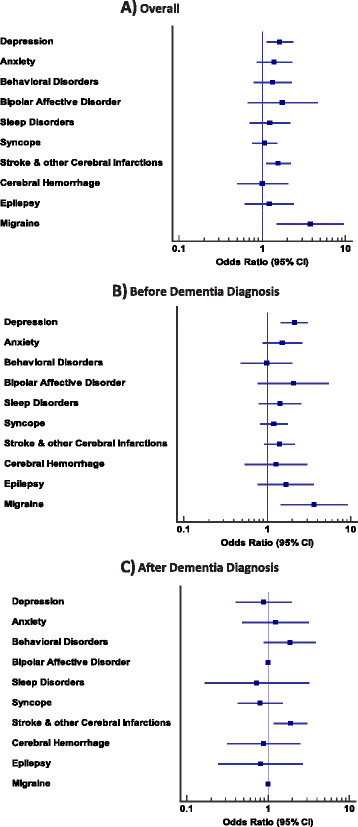
**Forest plot for odds ratios of the selected list of neuropsychiatric comorbidities for dementia with Lewy bodies compared with Alzheimer’s disease as the reference group in different three conditions. (A)** Overall assessment (regardless of the timing). **(B)** Comorbidities recorded prior to the diagnosis of dementia as a risk factor for type of dementia (DLB vs. AD). **(C)** Type of dementia (DLB vs. AD) as a risk factor for comorbidities occurred after the diagnosis of dementia. AD, Alzheimer’s disease; CI, confidence interval; DLB, dementia with Lewy bodies.

Figure 2 illustrates the forest plots of adjusted OR for each neuropsychiatric comorbidity in DLB with AD as the reference group. With respect to the timing of the events, depression (adjusted OR = 2.12 (95% CI = 1.49 to 3.03)) and migraine (adjusted OR = 3.65 (95% CI = 1.48 to 9.0)) were more commonly recorded before the diagnosis of dementia in the DLB group (Figure 2B). As shown in Figure 2C, ischemic stroke (adjusted OR = 1.89 (95% CI = 1.21 to 2.96)) was the only significant comorbid condition that was more likely to happen among the DLB patients compared with the AD population after the onset of dementia.

Further subgroup analysis was performed regarding gender, cognitive level and living place. As summarized in Table 6, depression and stroke were more common in the DLB group compared with AD patients among both males and females (all *P* < 0.05), whereas the higher prevalence of anxiety in DLB patients was statistically significant only among the males (3.4% vs. 1.8%, *P* = 0.031). With respect to level of cognition, depression, stroke and migraine were significantly more common in DLB patients compared with the AD group only among those with MMSE < 22 (all *P* < 0.05). Depression, sleep disorders and stroke were more commonly occurred in the DLB patients compared with the AD group among those who were living in their own house (all *P* < 0.05), while migraine was more prevalent in DLB patients who lived in nursing homes (2.7% vs. 0.2%, *P* = 0.045).

**Table 6:** **Frequency of different neuropsychiatric comorbidities in patients with dementia with Lewy bodies (*n*** **= 634) versus Alzheimer’s disease patients (*n*** **= 9,161) within different subgroups regarding gender, cognitive level at the time of diagnosis and living place using the chapters of the International Statistical Classification of Diseases and Related Health Problems, 10th Revision**

**Title**	**Gender**	**DLB (%)**	**AD (%)**	***P*** **value**	**Cognition**	**DLB (%)**	**AD (%)**	***P*** **value**	**Living place**	**DLB (%)**	**AD (%)**	***P*** **value**
Depression	Male	6.3	3.8	**0.020**	MMSE ≥ 22	6.3	5.2	0.350	Own house	7.0	4.5	**0.006**
	Female	8.7	4.8	**0.005**	MMSE < 22	7.4	3.5	**0.001**	Nursing home	9.3	3.9	0.068
Anxiety	Male	3.4	1.8	**0.031**	MMSE ≥ 22	3.3	2.7	0.464	Own house	3.2	2.6	0.372
	Female	3.2	3.0	0.901	MMSE < 22	3.3	2.7	0.528	Nursing home	4.0	2.8	0.469
Behavioral disorders	Male	3.4	2.6	0.381	MMSE ≥ 22	2.4	1.9	0.471	Own house	2.9	1.8	0.079
	Female	3.2	1.6	0.071	MMSE < 22	3.7	2.0	0.062	Nursing home	6.7	4.1	0.362
Bipolar affective disorders	Male	0.5	0.5	0.702	MMSE ≥ 22	0.6	0.5	0.689	Own house	0.9	0.4	0.185
	Female	1.2	0.5	0.129	MMSE < 22	1.1	0.4	0.105	Nursing home	0	1.0	1
Sleep disorders	Male	3.9	2.5	0.094	MMSE ≥ 22	2.7	2.0	0.339	Own house	2.7	1.6	**0.040**
	Female	0.8	1.1	1	MMSE < 22	2.2	1.1	0.124	Nursing home	2.7	1.4	0.325
Syncope	Male	7.1	6.7	0.789	MMSE ≥ 22	6.9	7.4	0.782	Own house	7.5	7.2	0.740
	Female	7.5	7.4	0.950	MMSE < 22	7.8	6.9	0.579	Nursing home	5.3	7.7	0.471
Stroke	Male	8.7	6.0	**0.043**	MMSE ≥ 22	6.9	5.3	0.193	Own house	8.4	5.1	**0.001**
	Female	7.9	4.8	**0.025**	MMSE < 22	9.3	5.0	**0.002**	Nursing home	8.0	7.1	0.772
Cerebral hemorrhage	Male	1.8	1.6	0.732	MMSE ≥ 22	1.2	1.2	0.547	Own house	1.8	1.4	0.410
	Female	1.2	1.3	1	MMSE < 22	1.9	1.7	0.807	Nursing home	0	1.8	0.613
Epilepsy	Male	1.6	1.5	0.958	MMSE ≥ 22	1.2	1.3	1	Own house	1.8	1.3	0.287
	Female	2.0	1.2	0.242	MMSE < 22	2.2	1.2	0.165	Nursing home	1.3	2.4	1
Migraine	Male	0.5	0.2	0.249	MMSE ≥ 22	0.9	0.4	0.165	Own house	0.7	0.3	0.138
	Female	1.6	0.4	0.580	MMSE < 22	1.1	0.2	**0.034**	Nursing home	2.7	0.2	**0.045**

Univariate comparisons were performed using either the chi-square or Fisher’s exact test wherever appropriate. Statistically significant differences (*P* < 0.05) in bold. AD, Alzheimer’s disease; DLB, dementia with Lewy bodies; MMSE, Mini-Mental State Examination.

## 4 Discussion

In this study we investigated selected risk factors and comorbidities in patients suffering from DLB in comparison with AD. There were more males in the DLB group, which is in line with previous studies [21]. At the time when dementia diagnosis was set, DLB patients were younger, and, despite similar level of cognitive impairment, lived more frequently in nursing homes and received more psychiatric medication and a higher total number of drugs. This indicates a worse health profile at the time when dementia was diagnosed and suggests that DLB patients may have been affected by a larger number of diseases before they developed dementia compared with the AD subjects. Patients with DLB were more frequently affected with depression, stroke and cerebrovascular infarctions and migraine.

### 4.1 Medication

There were some interesting differences in the use of medication. Cholinesterase inhibitors were used in the majority of DLB and AD patients. Although these drugs are indicated for AD and Parkinson’s disease with dementia, but not formally for DLB, there is good evidence that they are also useful in DLB [22],[23]. Memantine was prescribed for 15% of DLB patients and 10% of AD patients, although the evidence is less conclusive for DLB. However, there are some indications that memantine may in fact be useful also for DLB [24], including meta-analysis data [23].

### 4.2 Depression

Depression was more frequent in both men and women in the DLB group compared with AD, particularly in patients with a lower cognitive status at the time when dementia was diagnosed. Depression is a common feature of DLB [11], especially in its early stages. Several hypotheses have linked depression to the etiology and pathophysiology of dementia, depression may be a risk factor for DLB [9]. DLB has also been associated with a higher risk of depression [25]. The etiology of depression is probably multifactorial and the relation to dementia is complex. In our study, depression was significantly more often diagnosed in DLB patients before they were diagnosed with dementia. After the dementia diagnosis was set, depression occurrence did not differ between the DLB and AD patients.

It is a matter of dispute whether depression is a risk factor or a prodromal stage of DLB. Nonmotor symptoms and widespread brain pathological changes are believed to occur in DLB before dementia onset [26], so depression may be a sign of underlying pathological changes that are already present in DLB subjects. It would be of interest to investigate whether prevention or treatment of depression could decrease the incidence of DLB or postpone the development of dementia.

### 4.3 Migraine

In our study, migraine was more common in the DLB group before dementia was diagnosed. Furthermore, it occurred more frequently in DLB patients that had a lower MMSE score at the time of diagnosis. Migraine was shown as a risk factor for developing dementia [27] and associated with smaller brain tissue volumes [28]. Recently, headache has been suggested as a risk factor for the development of vascular dementia in a prospective population-based study [29]. However, there are no previous studies investigating migraine in DLB patients. Conditions that are common in DLB patients and subjects suffering from migraine include complex visual hallucination [30] and disorders in olfactory perception [31]. Even though their etiology seems different, further research on these two disorders could provide valuable insights into the relationship between DLB and migraine.

### 4.4 Stroke

Strokes were found to be more common for both men and women in the DLB group compared with the AD group, especially in subjects with lower MMSE scores. The occurrence of cerebrovascular infarctions but not cerebral hemorrhages after dementia diagnosis was significantly higher in the DLB group compared with AD patients. This relationship was not significant before dementia was diagnosed. This finding is surprising as strokes are common predictors for AD [32]. The difference in use of antipsychotics may be an explanation.

Studies on cerebrovascular pathology in DLB patients are not numerous and provide contradictory results. In a neuropathological study that included 96 DLB patients, no lesions of ischemic strokes were present in these subjects. The study suggested that the diagnosis of DLB had a protective effect against stroke [33]. Another study suggested that DLB patients with advanced typical Lewy body pathology were less likely to have history of stroke and cerebrovascular diseases [34]. This study revealed that infarcts, atherosclerosis and small vessel disease were inversely correlated to the extent of the DLB pathology. Nevertheless, cerebral amyloid angiopathy was associated with the grade of DLB pathology. A neuropathological case report revealed severe cerebral amyloid angiopathy in the presence of DLB pathology [35]. However, it can be argued that cerebral amyloid angiopathy is not related to DLB, but rather is a sign of pathological aging and concomitant AD [36],[37].

Even though the reasons are not clear, our study – strengthened by a large sample size – revealed a significantly frequent occurrence of stroke, specifically cerebral infarctions in DLB patients. More research is thus crucial to further elucidate this association. It can be speculated that the use of antipsychotics may have contributed to the development of strokes [38]. It is of interest that a considerable proportion of DLB patients were treated with antipsychotic medication before they were diagnosed with dementia (16%). This could be due to the clinical presentation of DLB, in which, in contrast to AD, frequent visual hallucinations are common early in the disease process.

Underlying psychiatric disorders may also be confounders [39] and lead to need for antipsychotics. It has been shown that antipsychotic treatment in dementia is associated with worse outcomes such as shorter time to nursing home admission, cerebrovascular events and mortality in older people [4],[40]-[42]. The Swedish guidelines for the treatment of neuropsychiatric symptoms in dementia therefore state that antipsychotics should be used very restrictively and only for psychotic symptoms or aggression that causes suffering or potential danger to the patient or others [20]. However, they are often prescribed to older people with dementia [43],[44], particularly in the nursing home setting [45],[46].

Cardiovascular comorbidities are frequent in all dementia disorders [47],[48] and belong to commonly reported causes of death [49]. In our study they have been found more common in the DLB group compared with AD, particularly in female patients, those who live in their own house and have a lower cognitive status at the beginning of the diagnostic process. A recent nationwide study in Sweden revealed that 60% of DLB patients were treated with cardiovascular medication, but the use of these drugs was lower in this group compared with AD [47]. However, this difference may be attributed to the fear of side effects in DLB patients; for example, side effects due to autonomic dysfunction. AD patients present the lowest mortality rate compared with other dementia disorders [7] and are therefore considered the healthiest group of dementia patients [50], even though there are some contradictory reports [51],[52]. Investigating comorbidities in patients with dementia can provide with valuable insights into dementia disorders and contribute to better understanding of their pathophysiology.

### 4.5 Limitations and strengths

One may criticize that the study is limited by the validity of diagnoses. However, the validity of the Swedish National Patient Registry has been shown to be high for many diagnoses [53]. Nevertheless, the underestimation of comorbidities is inevitable since the Swedish National Patient Registry is based on outpatient or inpatient referrals and those with mild symptoms who did not seek medical help are not recorded. The validity of the data in SveDem has been assessed, especially in memory clinics. The data registered in memory clinics in a random sample of patients were in good agreement with medical records in a validation process [54]. Moreover, although we considered timing for the events, the probability of reverse causation is not completely omitted.

The validity of the diagnosis of dementia disorders has not been examined. It is necessary to acknowledge that the way of diagnosing both dementia types in SveDem reflects clinical practice in Sweden, and biomarkers such as dopamine transporter single-photon emission computed tomography is not available at all centers. Symptoms of DLB and AD overlap, which leads to difficulties in the diagnostic process. Autopsy diagnosis is currently not available but many patients are followed longitudinally, which probably improves diagnostic accuracy. Linking SveDem to autopsy records in future could help assess the accuracy of the clinical diagnoses.

Our study benefits from one of the largest samples of DLB patients in the world. Most of the previous studies have focused either on a single or a small number of comorbidities, while we compared the entire comorbidity profile. Using the exact date of registration for each comorbidity and dementia, we had access to the consecutive timing of the events in order to determine whether comorbidities occurred either before or after dementia diagnosis. Both SveDem and the Swedish National Patient Registry have just a minute proportion of missing values. The personal registration number makes it possible to follow each individual over time and to connect corresponding information from different registries in Sweden. The number of hospital stays with missing personal registration numbers in the inpatient registry was only 0.6% in 2006 [53].

## 5 Conclusions

Our study indicated a worse comorbidity profile in DLB patients, with a higher prevalence of depression, stroke and migraine, compared with the AD population. Deeper knowledge about these differences among the DLB and AD groups is needed. Future studies could explore whether the presented associations are due to different mechanisms of these disorders as well as studying their influence on diagnostics and care.

## Abbreviations

AD: Alzheimer’s disease

CI: confidence interval

DLB: dementia with Lewy bodies

ICD: International Classification of Diseases

MMSE: Mini-Mental State Examination

OR: odds ratio

SD: standard deviation

SveDem: Swedish Dementia Registry

## Competing interests

The authors declare that they have no competing interests.

## Authors’ contributions

S-MF was involved in the conception, design and acquisition of data and carried out analysis, interpreted the results and wrote the manuscript. SD contributed to the conception and carried out data mining, analysis and writing of the manuscript. PC participated in the interpretation of the findings, writing of the manuscript and substantial contribution to its content. DA participated in the interpretation of the findings and revised the manuscript critically for important intellectual content. ME conceived of the study, and contributed to its design and coordination and to critical improvement of the manuscript. DR made substantial contributions to conception, design and acquisition of data, participated in the interpretation of the findings and revised the manuscript critically. All authors read and approved the final manuscript.
